# Apatinib inhibits the proliferation of gastric cancer cells via the AKT/GSK signaling pathway *in vivo*

**DOI:** 10.18632/aging.203458

**Published:** 2021-08-27

**Authors:** Yi Chen, Nan Chen, Jin Xu, Xindong Wang, Xiaowei Wei, Cuiju Tang, Zhong Duanmu, Junfeng Shi

**Affiliations:** 1 Department of Oncology, Nanjing Pukou Central Hospital, Pukou Branch Hospital of Jiangsu Province Hospital, Nanjing 210000, China; 2 Department of Outpatient, General Hospital of Eastern Theater Command, PLA, Nanjing 210002, China; 3 Department of Thyroid and Mammary Gland Surgery, Nanjing First Hospital, Nanjing Medical University, Nanjing 210000, China; 4 Department of Oncology, Medical School, Southeast University, Nanjing 210009, China; 5 Department of Oncology, Nanjing First Hospital, Nanjing Medical University, Nanjing 210000, China

**Keywords:** apatinib, gastric cancer, AKT/GSK signaling, angiogenesis, proliferation

## Abstract

Gastric cancer (GC) is the third leading cause of cancer-associated mortality globally. Although the diagnosis and therapeutic strategies for GC have improved, the prognosis for advanced gastric cancer (AGC) remains poor. Hence, the present study sought to design a zebrafish model established by microinjecting human MGC-803 GC cell line for studying personalized molecular-targeted cancer therapy. Apatinib, a novel molecular-targeted agent, was evaluated for its *in vivo* efficacy through a comparison among the control groups (no treatment) and subject groups (treatment). Newly formed vessel length and tumor volume were measured in all of the groups for further study. The length of newly formed vessels was obviously shortened after apatinib treatment in the zebrafish model established in this study. Meanwhile, apatinib exhibited the best antitumor growth effect with dose and time dependence by suppressing AKT/GSK3α/β signaling, which may be the mechanism underlying the profound antitumor clinical effect of apatinib. The data indicated that apatinib therapy exerts an anti-angiogenesis effect and it can be recommended as a proper antitumor growth therapy for GC patients. Additionally, zebrafish models could be designed as a potential practical tool to explore new anti-GC cancer drugs.

## INTRODUCTION

Gastric cancer (GC) is the third leading cause of cancer-associated mortality globally and approximately 90% of GCs are diagnosed as adenocarcinoma, presenting a high mortality rate [1]. Although multimodal diagnosis and treatment measures have been developed, treatment for advanced gastric cancer (AGC) remains palliative and overall survival rates have remained poor during the last four decades [2]. Alternative treatments become scarce once post-surgical recurrence or therapeutic failure of classic first-line combinations occur [3, 4]. Clinical effects of conventional chemotherapy and radiotherapy for GC can be different considering the age, stages, molecular subtype and comorbidities [5]. However, the application of molecular-targeted anticancer drugs (e.g., trastuzumab and apatinib) provides a promising optional therapy for GC, especially AGC [6].

Apatinib, known as YN968D1 (molecular weight 493.58 Da), is one of the most efficient oral anticancer agents, verified by preclinical and clinical trials for the treatment of various solid malignancies [7–9]. This novel small-molecule drug targets angiogenesis by selective inhibition of vascular endothelial growth factor receptor-2 (VEGFR-2) and it can partly inhibit c-Kit and c-Src tyrosine kinases [10]. Some standard clinical trials have reported that apatinib can prolong the overall survival (OS) and progression-free survival (PFS) of GC individuals with tolerable safety profiles [11, 12]. Meanwhile, some studies, conducted in xenograft mouse models, showed that apatinib can enhance apoptotic cell death and inhibit final solid tumor volume with tolerable adverse reactions when applied alone or in combination with other chemotherapeutic drugs [13–14]. However, there are no practical biomarkers that can hint toward the most significant application of apatinib in GC patients during clinical practice.

The zebrafish (*Danio rerio*) is an increasingly popular and powerful animal model applied in the field of cancer biology. Its unique advantages, including small size, optical transparency, breeding convenience, and high throughput, make xenograft observation easier and more precise [15, 16]. Additionally, its high level of genetic homology to humans provides a priority to be a versatile organism for exploration of cancer development and pharmacological mechanism [17, 18]. Hence, it can serve as a practical pre-clinical or clinical model to personalize anticancer therapy.

In the present study, we generated a zebrafish model utilizing human GC cell lines and then described the potential anticancer efficacy of apatinib in this model. Moreover, these data indicated that apatinib has anti-neoplastic and anti-angiogenic activities through inhibition of AKT/GSK3α/β signaling in GC cells. Most importantly, this original research can provide a reliable platform for evaluation of the applicability and efficacy of apatinib, even for personalizing GC therapy in clinical practice in the future.

## MATERIALS AND METHODS

### Cell culture and reagents

Human GC cell lines, including MGC-803, HGC-27, AGS, BGC-823, and SGC-7901, were obtained from the Cell Bank of Chinese Academy of Medical Science (Shanghai, China) and cultured in Dulbecco’s modified Eagle’s medium(DMEM). Cells were cultured in the incubator at 37°C with 5% CO_2_ (v/v). Besides, apatinib (Jiangsu HengRui Medicine Co., Ltd.) was dissolved in DMSO and stocked in different concentrations for further study. SC79, an AKT activator, was purchased from Beyotime Biotechnology, Shanghai, China.

### Cell viability assay

GC cells were seeded at 5000 cells per well into 96-well plates, and then treated with the indicated concentrations of apatinib (8 h and 72 h). After that, cells were treated with 10 μl MTT (5 mg/ml) at 37°C for 4 hours followed by 150 μl dimethylsulphoxide and determined by measuring the absorbance at 570 nm using a microplate reader (Bio-Rad, USA).

### Western blot analysis

Total protein was extracted from the cultured cells, and then subjected to 10% SDS-polyacrylamide gel electrophoresis and transferred onto 0.45 μm PVDF membranes (Millipore). Membranes were then blocked in 5% milk-TBST for 1 h and incubated with primary antibodies p-AKT, p-GSK3α, p-GSK3β (diluted 1:1000), and AKT, GSK3α, GSK3β, β-actin (diluted 1:5000 in TBST) overnight. After appropriate secondary antibodies for 1 h at room temperature, the membranes were detected by Tanon 5200 Imaging System (Shanghai, China). Antibodies were purchased as follows: p-AKT (Abcam, Cat. ab81283), AKT (Abcam, Cat. ab179463), p-GSK3α (Abcam, Cat. ab131112), GSK3α (Abcam, Cat. ab40870), p-GSK3β (Abcam, Cat. ab75814), GSK3β (Abcam, Cat. ab32391).

### Preparation of a transgenic zebrafish line

A fluorescent transgenic zebrafish line expressing fli1a: EGFP was obtained from the Model Animal Research Center of Nanjing University. The zebrafish line was maintained at 28.5°C according to the standard zebrafish husbandry protocols, as previously described [19, 20]. Human MGC-803 cell line was fluorescently labeled with CM-DiI (Invitrogen, Life Technologies, Carlsbad, CA, USA) for 30 min according to the manufacturer’s instructions.

### Disposal of zebrafish embryo

At 48 hours post-fertilization (hpf), transgenic zebrafish lines were anesthetized with tricaine (Sigma-Aldrich, St. Louis, MO, USA) and mounted on an agarose pad. The dyed MGC-803 cells (labeled with CM-Dil) were mixed with 5% PVC medium until the cell density reached 3 × 10^7^ cells/ml, and then they were injected into the yolk sac of every single zebrafish embryo using a microinjector (IM-31, Narishige, Japan) under observation by a stereoscope (SMZ 745 T, Nikon, Japan). The injection volume was 10 nl. We selected zebrafish with a successful injection and relatively homogenous tumor cell size as the experiment object. This zebrafish research was assisted by the School of Pharmaceutical Sciences of Nanjing Technologic University.

### Apatinib treatment of xenograft tumors and imaging

The dyed MGC-803 cells were inoculated in the fluorescent transgenic zebrafish lines tg (fli1a: EGFP). After developing a palpable mass, zebrafish were randomized to either the apatinib treatment group or the control group (>30 per group). As previously described, different concentrations of apatinib were dissolved in breeding water. Zebrafish were cultivated in breeding water at 32°C avoiding light box. After 1 day of administration, the subintestinal vessels (SIVs) of zebrafish embryos (>60 per group) in all of the treatment groups were photographed by a fluorescence microscope, and abnormal branch length and area of SIVs were quantified using the count/size function of Image-Pro Plus 6.0 software. The total area of SIVs was quantified in pixels. The drug effect was calculated using the following formula: SIV inhibition. We monitored tumor cell growth *in vivo* at 1, 2 and 3 days post treatment (dpt) by an inverted fluorescence microscope (IX71, Olympus, Japan). Angiogenesis was observed by a confocal microscope (LSM710, ZEISS, Germany). After 3 days of administration, the number of tumor cells in zebrafish was calculated in both the control and subject groups.

### Statistical analysis

The SPSS ver. 18.0 (SPSS Inc., Chicago, IL) was used for analysis of all data. The results are expressed as mean ± SD. Multiple group data and multiple comparisons were analyzed by one-way ANOVA. A *P*-value of less than 0.05 was considered to be statistically significant for all of the analyses. (^**^) indicated statistical significance *P* < 0.01, (^*^) *P* < 0.05.

## RESULTS

### Inhibition effect of apatinib on the cell viability in different gastric cancer cell lines

**Figure 1:**
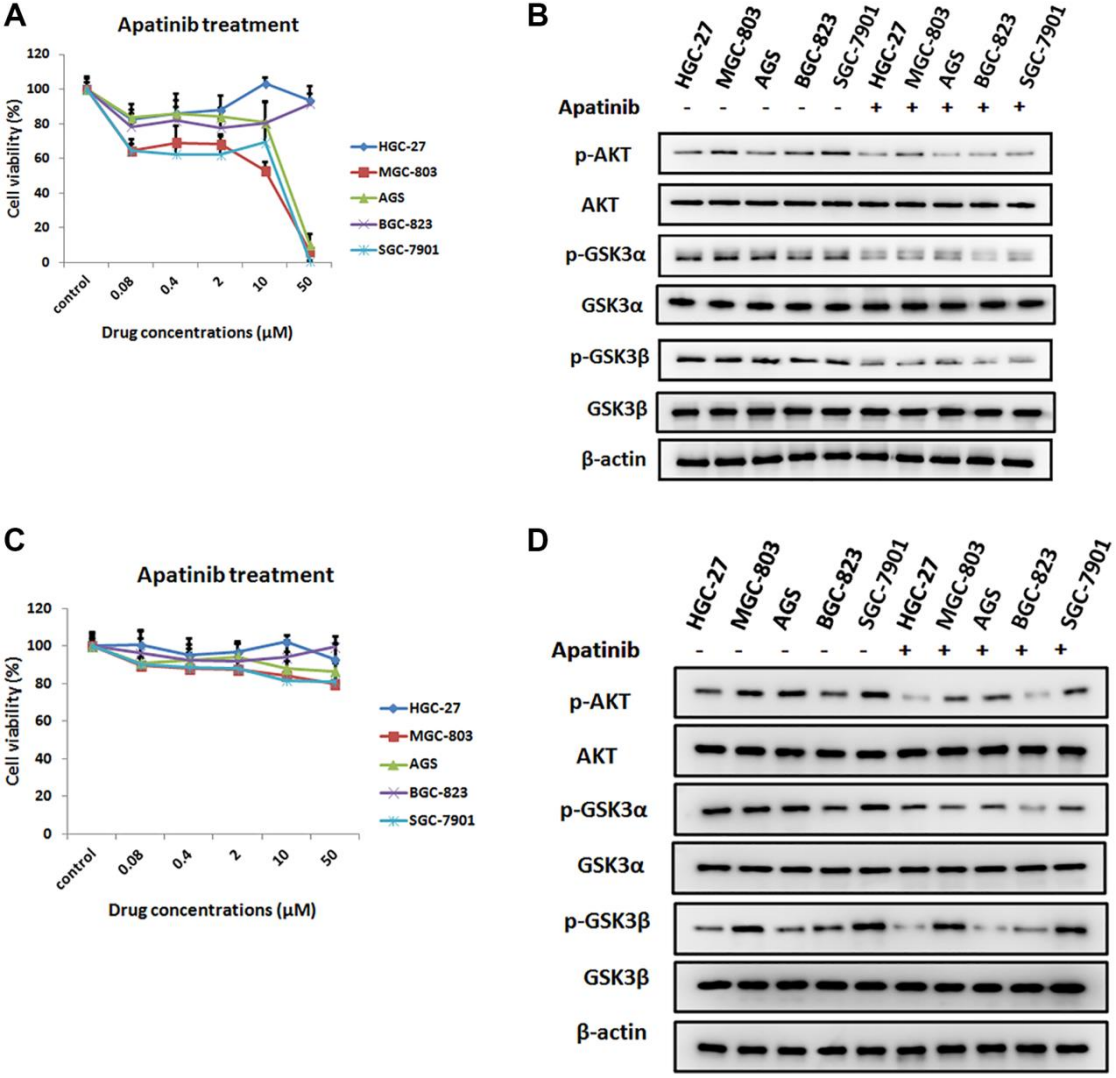
**The inhibition effect of apatinib on the cell viability in different GC cell lines.** (**A**) Cell viability of different human gastric cancer cell lines treated with different concentrations of apatinib for 72 h. (**B**) After treatment with apatinib 2 μM concentration for 72 h, western blot analysis of AKT, phosphorylated AKT (p-AKT), GSK3α, phosphorylated GSK3α (p-GSK3α), GSK3β and phosphorylated GSK3β (p-GSK3β) in GC cell lines. (**C**) Cell viability of different human gastric cancer cell lines treated with different concentrations of apatinib for 8 h. (**D**) After treatment with apatinib 2 μM concentration for 8 h, western blot analysis of AKT, phosphorylated AKT (p-AKT), GSK3α, phosphorylated GSK3α (p-GSK3α), GSK3β and phosphorylated GSK3β (p-GSK3β) in GC cell lines.

To study the effect of apatinib on gastric cancer cells, *in vitro* cytotoxicity experiments of apatinib were performed on different GC cell lines. As shown in Figure 1A, all of the GC cell lines were treated with apatinib at different concentrations from 0.02 μmol/L to 50 μmol/L (μM) for 72 h. The results show that apatinib could not evidently inhibit the proliferation of GC cells in the *in vitro* experiments, especially in HGC-27 and BGC-823 cell lines. However, when the dose reached 50 μM, three kinds of GC cells were efficiently suppressed, namely, MGC-803, AGS, and SGC-7901. These data indicated that apatinib demonstrated cytotoxicity to GC cells *in vitro* assay.

Previous research has found that AKT/GSK-3β/β-catenin signaling is crucial for the proliferation and invasion of gastric cancer cells [21]. To further elucidate the mechanism of the inhibition effect of apatinib, we detected the levels of AKT and GSK in different GC cell lines, which were pretreated with 2 μM apatinib for 72 hours. It was observed that apatinib could inhibit the phosphorylation of AKT, GSK-3α and GSK-3β in different GC cell lines to varying degrees, which could be due to induction of cell death (Figure 1B). The same result can be found after pretreatment with apatinib for 8 hours (Figure 1C, 1D). Thus, the finding suggested that apatinib may induce apoptosis through inhibition of the expression levels of p-AKT and p-GSK protein.

### Cell line MGC-803 promoted angiogenesis in zebrafish embryos

**Figure 2:**
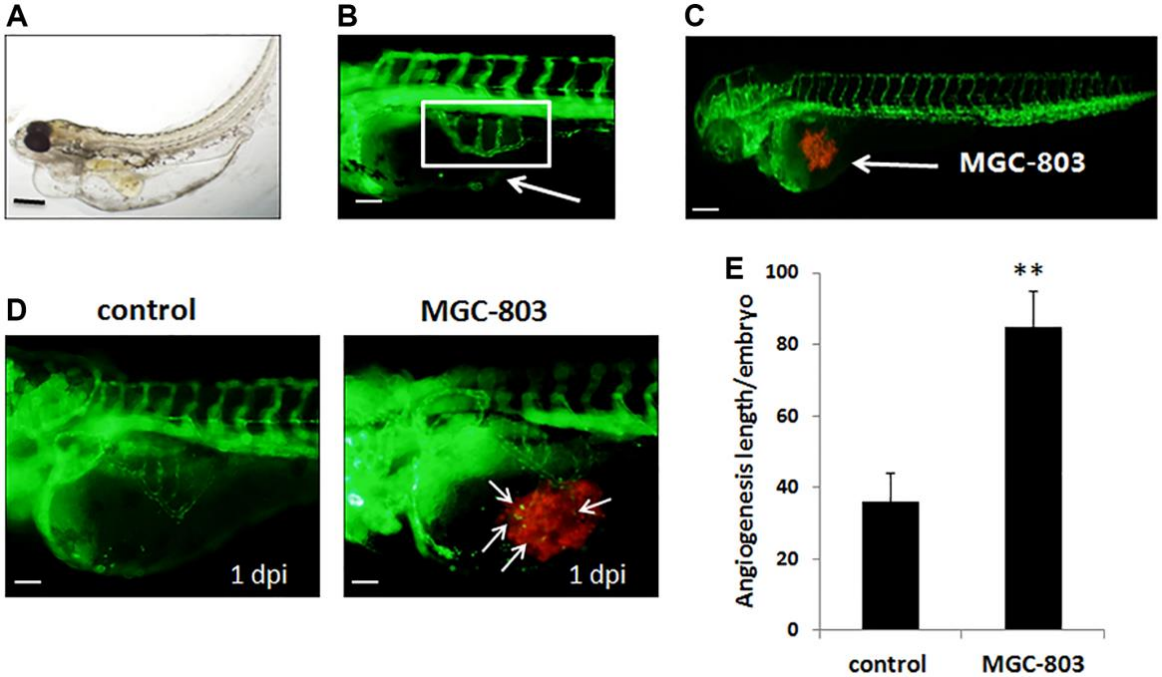
**Gastric cancer cell MGC-803 promoted angiogenesis in transgenic zebrafish tg (fli1a-EGFP).** (**A**) Biologic morphology of zebrafish. Scale bar: 200 μm. (**B**) Fluorescence image of subintestinal vessels of the uninjected embryo. Scale bar: 50 μm. (**C**) Gastric cancer cell lines were injected to the zebrafish embryo, MGC-803 was taken as an example in the figure. Scale bar: 200 μm. (**D**) MGC-803 cells promoted angiogenesis at 1 dpi compared with control group. The white arrow indicated the tumor cell induced angiogenesis, dpi: days post injection. Scale bar: 50 μm. (**E**) Quantitative analysis of the length of newly formed vessels in zebrafish with/without inducing by MGC-803.

To further investigate the antitumor effect of apatinib, *in vivo* studies were conducted in the zebrafish model system. During the time frame from 48 hpf, the intact SIVs of transgenic zebrafish tg embryos were formed and they looked like a basket (Figure 2A, 2B). All of the injected GC cell lines caused proangiogenic behavior in zebrafish embryos as early as 1 dpi (Figure 2C), and the SIVs of the embryos formed additional branches and extended toward the tumor implantation mass (Figure 2D). As shown in Figure 2E, the length of angiogenesis in this zebrafish embryo model was significantly increased.

### Maximum safe dose of apatinib for the zebrafish xenografts model

**Figure 3:**
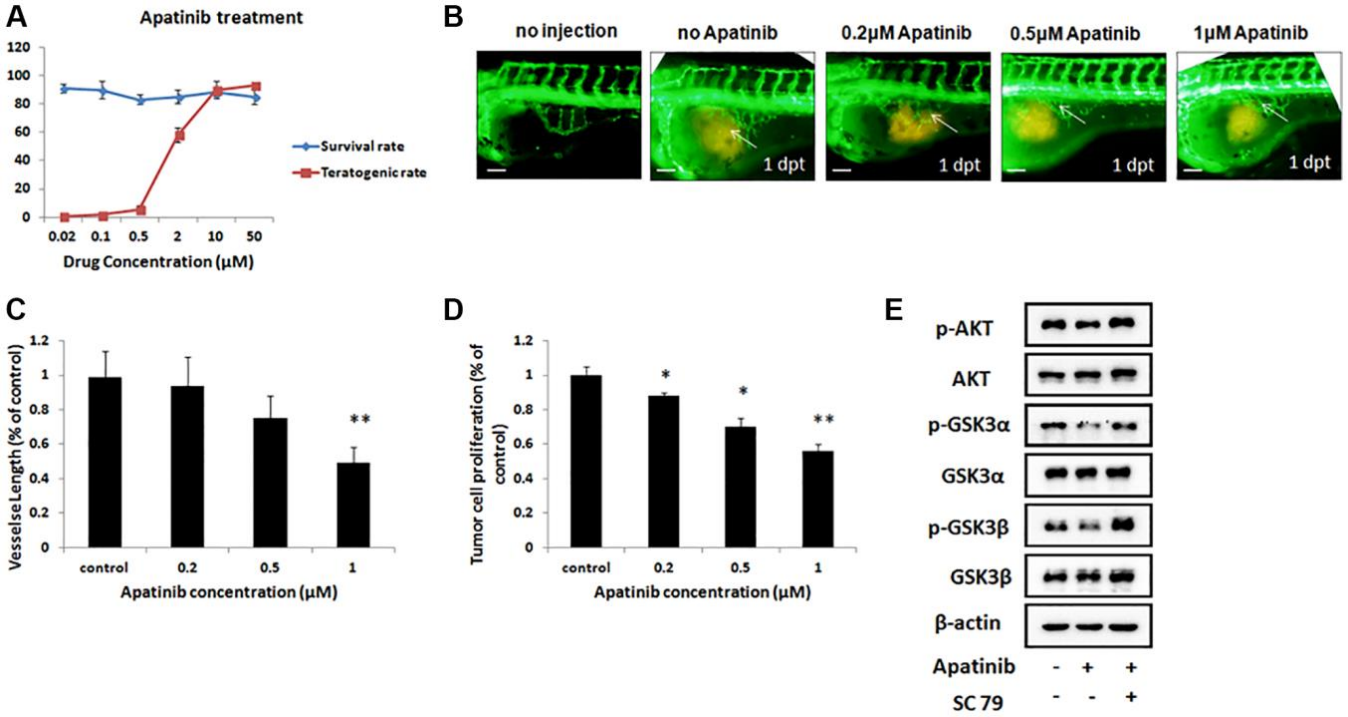
**Inhibitory effect of gastric cancer cell line in the zebrafish xenograft model under apatinib treatment.** (**A**) The survival rates and teratogenic rates of zebrafish were detected under different concentrations of apatinib. (**B**) Fluorescence image of subintestinal vessels in zebrafish induced by MGC-803 with/without apatinib treatment at 1 dpt. (**C**) Quantitative analysis of the length of newly formed vessels in zebrafish induced by MGC-803 with/without apatinib treatment. Scale bar: 50 μm. (**D**) Quantitative analysis of the MGC-803 cells proliferation with/without apatinib treatment at 2 dpt. Dpt: days post treatment. (**E**) Western blot analysis of AKT, phosphorylated AKT (p-AKT), GSK3α, phosphorylated GSK3α (p-GSK3α), GSK3β and phosphorylated GSK3β (p-GSK3β) in injected cells.

Moreover, to identify the safe dose of apatinib therapy for GC, transgenic zebrafish (fli1a-EGFP), grafted with a human GC cell line MGC-803, were treated with apatinib at different concentrations from 0.02 μM to 50 μM. As apatinib administered by soaking, drug exposure by addition to the breeding water. First, we measured the survival rates and teratogenic rates in the zebrafish models after drug administration, which are an indicator of the side effects of apatinib treatment. As shown in Figure 3A, all of the treatments at diverse concentrations did not alter the survival rate of the zebrafish. However, apatinib treatment steeply increased the teratogenic rate in the zebrafish when the dose was more than 2 μM, suggesting a significant adverse effect of this dose. This result also showed that the proper therapeutic effect of apatinib treatment should be below 2 μM. When treated with apatinib at 0.5 μM, 50% of the zebrafish developed pericardial edema. As the concentration increased, zebrafish developed morphological abnormalities, such as decreased eye size, shortened body, and axis bending.

### Inhibition effect of apatinib on the angiogenesis in the zebrafish xenograft model

As described previously, apatinib can be applied in the zebrafish xenograft model at a dose below 2 μM without any obvious teratogenesis and death. Therefore, we decided to investigate the difference in the length of newly formed vessels in the zebrafish treated with apatinib, whose concentration was selected from 0.2 μM to 1 μM.

Angiogenesis is critical during tumor progression. To examine whether these different concentrations therapies of apatinib inhibit the formed vessels in this zebrafish xenograft model, which were induced into MGC-803 cells, we evaluated the length by quantitative analysis of the newly formed vessels using a fluorescence microscope and Image-Pro Plus 6.0 software. As shown in Figure 3B, the length of the newly formed vessels was significantly reduced by treatment with 1 μM apatinib with 1 dpt. In addition, apatinib treatment showed a dose dependent reduction effect on all measured parameters when compared with the control group (Figure 3C). These data indicated that apatinib was effective in inhibiting the newly formed vessels of GC.

### Inhibition effect of apatinib on tumor cell proliferation in the zebrafish xenograft model

We further studied whether these doses of apatinib affect the proliferation of MGC-803 in this zebrafish model. As shown in Figure 3D, apatinib treatments at 2 dpt showed a marked reduction effect on cell proliferation when compared with the control group. This result was in line with our previous data, elucidating that apatinib treatment showed a dose-dependent effect in inhibiting cancer cell proliferation, which might be caused by angiogenesis in the tumor cell microenvironment.

To further ascertain whether the inhibition of apatinib *in vivo* alters the AKT signal pathway, we investigated the phosphorylated AKT and GSK. As shown in Figure 3E, apatinib could inhibit the phosphorylation of AKT and GSK in injected cells. Furthermore, SC79, an AKT activator, could attenuate the effect of apatinib. Taken together, our data suggest that apatinib inhibits cell growth through the suppression of phosphorylation of AKT/GSK3α/β in GC cells.

**Figure 4:**
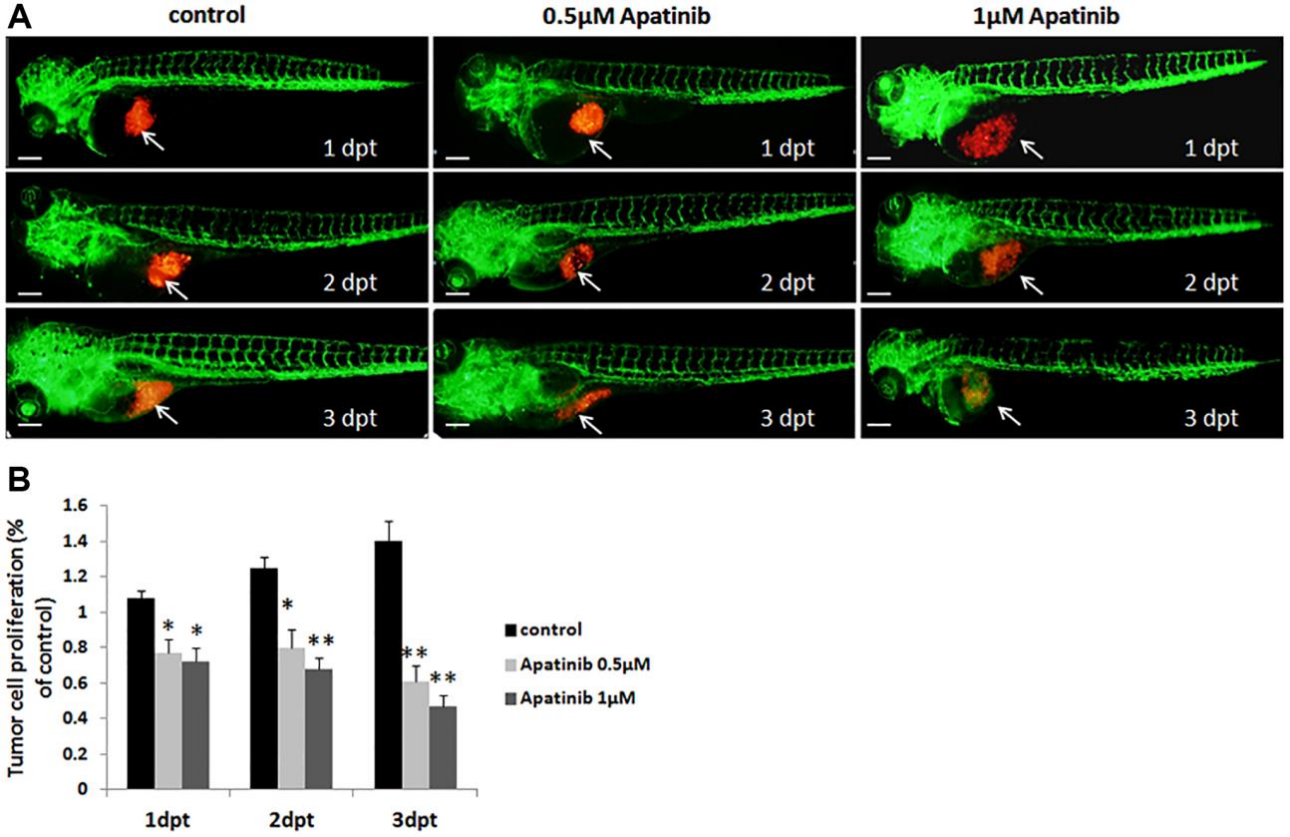
**Proliferation of zebrafish xenograft model inhibited by apatinib treatment.** (**A**) Fluorescence image of tumor size in zebrafish induced by MGC-803 with/without apatinib treatment at 1, 2 and 3 dpt. Scale bar: 200 μm. (**B**) Quantitative analysis of the length of MGC-803 cells proliferation in zebrafish with/without apatinib treatment. Dpt: days post treatment.

To clarity the time-effect relationship between cancer cell proliferation and apatinib concentrations, we measured MGC-803 cell proliferation after treatment with 0.5 μM apatinib at 1, 2, and 3 dpt, respectively (Figure 4A). As shown in Figure 4B, apatinib significantly decreased tumor cell proliferation in the zebrafish model implanted with MGC-803 cells in a time-dependent manner. These data were again in accordance with our previous observation in which apatinib was found to be an effective therapy for tumor cell proliferation. Furthermore, the zebrafish model established in this study has specific features that makes it an ideal candidate for screening anti-tumor drugs.

## DISCUSSION

Apatinib, a highly selective tyrosine kinase inhibitor, has shown promising antitumor effects on malignant tumors. Here, we designed and established a GC zebrafish xenograft model for evaluation of clinical effects of apatinib in GC patients. In the present study, our data showed that apatinib inhibited tumor progression by negatively regulating the AKT/GSK3α/β pathway in GC. The PI3K/Akt signaling pathway plays an important role in a variety of tumors, including GC, and it is closely related to tumorigenesis, proliferation, invasion, apoptosis, and autophagy. We found that the newly formed vessel length in the gastric cancer and tumor growth in the groups that were treated by apatinib at several concentrations could be commonly considerably inhibited in a dose- and time- dependent manner when compared with the control group, which was shown in the previous study to act via inhibiting the AKT/GSK3α/β signaling pathway. This findings was consistent with previous studies on human colon cancer, osteosarcoma and thyroid cancer [22–24]. This positive result might be a hint for further researches related to the relationships between particular molecule-targeted drugs and precision medicine.

Although GC is one of the common carcinomas arising from the digestive system, approximately two-thirds of the individuals are preliminarily diagnosed with AGC and unfortunately, they might miss radical surgical treatments or traditional chemotherapy-based therapy [25, 26]. The regular outcomes of conventional combination chemotherapies, including capecitabine, oxaliplatin, and 5-FU, remain poor, manifesting that the average OS of these patients is no more than 12 months and the 5-year survival value is under 10% [27, 28]. Nevertheless, the emergence of biologic therapies provides another effective and tailored choice. At present, targeted therapies approved for treating GC include trastuzumab, ramucirumab and nivolumab or pembrolizumab [29, 30]. A large-scale, real-world study demonstrated that apatinib had a favorable effectiveness and safety profile in patients with advanced gastric cancer [31]. Therefore, apatinib has also been approved as a targeted treatment for advanced gastric cancer.

It has been reported that Wnt/β-catenin, Jak-STAT, JNK and ERK signaling pathways can regulate the progression of GC [32, 33]. Moreover, the PI3K/AKT/mTOR signaling pathway plays a pivotal role in primary and acquired resistance in GC [34]. A study by H. Jiang et al. showed that the activation of Akt inhibits GSK3β activity, subsequently suppressing the phosphorylation of Snail, inducing Snail protein stabilization and nuclear localization, which ultimately promotes EMT [35]. In addition, a study has shown that apatinib can inhibit the metastasis of cancer via EMT inhibition [36]. In this study, our data showed that the protein expressions of p-Akt, p-GSK3α and p-GSK3β were decreased after apatinib treatment of MGC803 cells. Besides, Akt agonist SC79 was used to reverse the inhibition of AKT/GSK3α/β mediated by apatinib, demonstrating that apatinib was able to inhibit the AKT/GSK3α/β signaling pathway and thus induced apoptosis. In conclusion, our study for the first time confirmed apatinib’s anti-tumor effect through PI3K/Akt/GSK signaling pathway *in vitro* and *in vivo*, enriching the anti-tumor mechanism of targeted drug apatinib.

A large number of researches have reported the molecular mechanisms and *in vivo* effects of GC treatments, but most of them were performed in experimental mouse models [37, 38]. In our study, a GC zebrafish model was utilized to describe the potential efficacy of apatinib at different concentrations, which presents the advantages and values. First , many proven oncogenes and tumor suppressor genes have zebrafish homologs, as well as significant signaling pathways relevant to cancer cell proliferation, differentiation, migration and apoptosis [39, 40]. The pioneers of human tumor xenotransplantation in a zebrafish model were Lee et al. [41], who confirmed that human melanoma cell lines continued to have their own biological functions when injected into the zebrafish. These basic characterizations guarantee the rationality and reliability of human-disease-related zebrafish models. Second, the small size of zebrafish enables them to be raised in isolated well plates (e.g, 12-well, 24-well), providing a high- or medium-throughput measure which is considerably faster and less costly than mouse-injected models [41]. Moreover, the transparency of zebrafish embryos makes it easier to observe and quantify them. In parallel with the nature advantages of zebrafish models, a toolkit of experimental measures, including reverse genetic technique, makes it possible to study and optimize experimental zebrafish models [42]. As highlighted above, zebrafish models with human tumor xenotransplantation have a promising future in the field of cancer therapeutic research.

As already known, cancer treatments are evolving into precision medicine, aiming at personalizing therapy at the molecular level and achieving the best prognosis. With the assistance of the newly generated zebrafish models, it could be more convenient and intuitionistic to observe the curative effect of apatinib and other small-molecule targeted agents. Besides, the application of this innovative animal model will set the patients free from molecular genotyping tests, which improves the drug screening system such that it becomes time-saving and less costly.

## References

[1] Khazaei S, Rezaeian S, Ayubi E, Gholamaliee B, Pishkuhi MA, Khazaei S, Mansori K, Nematollahi S, Sani M, Hanis SM. Global Prostate Cancer Incidence and Mortality Rates According to the Human Development Index. Asian Pac J Cancer Prev. 2016; 17:3793–96. 27644618

[2] Petrioli R, Francini E, Laera L, Fiaschi AI, Ponchietti R, Roviello G. Role of chemotherapy in the treatment of metastatic castration-resistant prostate cancer patients who have progressed after abiraterone acetate. Cancer Chemother Pharmacol. 2015; 76:439–45. 10.1007/s00280-015-2803-y 26082421

[3] Al-Batran SE, Van Cutsem E, Oh SC, Bodoky G, Shimada Y, Hironaka S, Sugimoto N, Lipatov ON, Kim TY, Cunningham D, Rougier P, Muro K, Liepa AM, et al. Quality-of-life and performance status results from the phase III RAINBOW study of ramucirumab plus paclitaxel versus placebo plus paclitaxel in patients with previously treated gastric or gastroesophageal junction adenocarcinoma. Ann Oncol. 2016; 27:673–79. 10.1093/annonc/mdv625 26747859 PMC4803452

[4] Hara H, Kadowaki S, Asayama M, Ooki A, Yamada T, Yoshii T, Yamaguchi K. First-line bolus 5-fluorouracil plus leucovorin for peritoneally disseminated gastric cancer with massive ascites or inadequate oral intake. Int J Clin Oncol. 2018; 23:275–80. 10.1007/s10147-017-1198-7 29039072

[5] Zhang M, Deng W, Cao X, Shi X, Zhao H, Duan Z, Lv B, Liu B. Concurrent apatinib and local radiation therapy for advanced gastric cancer: A case report and review of the literature. Medicine (Baltimore). 2017; 96:e6241. 10.1097/MD.0000000000006241 28248891 PMC5340464

[6] Li K, Li J. Current Molecular Targeted Therapy in Advanced Gastric Cancer: A Comprehensive Review of Therapeutic Mechanism, Clinical Trials, and Practical Application. Gastroenterol Res Pract. 2016; 2016:4105615. 10.1155/2016/4105615 26880889 PMC4736909

[7] Zhang H. Apatinib for molecular targeted therapy in tumor. Drug Des Devel Ther. 2015; 9:6075–81. 10.2147/DDDT.S97235 26622168 PMC4654530

[8] Geng R, Li J. Apatinib for the treatment of gastric cancer. Expert Opin Pharmacother. 2015; 16:117–22. 10.1517/14656566.2015.981526 25420417

[9] Yu M, Gao Z, Dai X, Gong H, Zhang L, Chen X, Zhong DF, Sy SK. Population Pharmacokinetic and Covariate Analysis of Apatinib, an Oral Tyrosine Kinase Inhibitor, in Healthy Volunteers and Patients with Solid Tumors. Clin Pharmacokinet. 2017; 56:65–76. 10.1007/s40262-016-0427-y 27379402

[10] Tian S, Quan H, Xie C, Guo H, Lü F, Xu Y, Li J, Lou L. YN968D1 is a novel and selective inhibitor of vascular endothelial growth factor receptor-2 tyrosine kinase with potent activity in vitro and in vivo. Cancer Sci. 2011; 102:1374–80. 10.1111/j.1349-7006.2011.01939.x 21443688 PMC11158267

[11] Li J, Qin S, Xu J, Guo W, Xiong J, Bai Y, Sun G, Yang Y, Wang L, Xu N, Cheng Y, Wang Z, Zheng L, et al. Apatinib for chemotherapy-refractory advanced metastatic gastric cancer: results from a randomized, placebo-controlled, parallel-arm, phase II trial. J Clin Oncol. 2013; 31:3219–25. 10.1200/JCO.2013.48.8585 23918952

[12] Li J, Qin S, Xu J, Xiong J, Wu C, Bai Y, Liu W, Tong J, Liu Y, Xu R, Wang Z, Wang Q, Ouyang X, et al. Randomized, Double-Blind, Placebo-Controlled Phase III Trial of Apatinib in Patients With Chemotherapy-Refractory Advanced or Metastatic Adenocarcinoma of the Stomach or Gastroesophageal Junction. J Clin Oncol. 2016; 34:1448–54. 10.1200/JCO.2015.63.5995 26884585

[13] Peng QX, Han YW, Zhang YL, Hu J, Fan J, Fu SZ, Xu S, Wan Q. Apatinib inhibits VEGFR-2 and angiogenesis in an *in vivo* murine model of nasopharyngeal carcinoma. Oncotarget. 2017; 8:52813–22. 10.18632/oncotarget.17264 28881773 PMC5581072

[14] Peng H, Zhang Q, Li J, Zhang N, Hua Y, Xu L, Deng Y, Lai J, Peng Z, Peng B, Chen M, Peng S, Kuang M. Apatinib inhibits VEGF signaling and promotes apoptosis in intrahepatic cholangiocarcinoma. Oncotarget. 2016; 7:17220–29. 10.18632/oncotarget.7948 26967384 PMC4941382

[15] Nüsslein-Volhar C, Dahm R. Zebrafish: A Practical Approach. 2002. Oxford : Oxford University Press.

[16] Hong S, Lee P, Baraban SC, Lee LP. A Novel Long-term, Multi-Channel and Non-invasive Electrophysiology Platform for Zebrafish. Sci Rep. 2016; 6:28248. 10.1038/srep28248 27305978 PMC4910293

[17] Snaar-Jagalska BE. ZF-CANCER: developing high-throughput bioassays for human cancers in zebrafish. Zebrafish. 2009; 6:441–43. 10.1089/zeb.2009.0614 19954326

[18] Barriuso J, Nagaraju R, Hurlstone A. Zebrafish: a new companion for translational research in oncology. Clin Cancer Res. 2015; 21:969–75. 10.1158/1078-0432.CCR-14-2921 25573382 PMC5034890

[19] Cantas L, Sørby JR, Aleström P, Sørum H. Culturable gut microbiota diversity in zebrafish. Zebrafish. 2012; 9:26–37. 10.1089/zeb.2011.0712 22428747 PMC3308716

[20] Hensley MR, Leung YF. A convenient dry feed for raising zebrafish larvae. Zebrafish. 2010; 7:219–31. 10.1089/zeb.2010.0652 20441525

[21] Chen L, Xu M, Zhong W, Hu Y, Wang G. Knockdown of DDX46 suppresses the proliferation and invasion of gastric cancer through inactivating Akt/GSK-3β/β-catenin pathway. Exp Cell Res. 2021; 399:112448. 10.1016/j.yexcr.2020.112448 33347858

[22] Meng X, Wang H, Zhao J, Hu L, Zhi J, Wei S, Ruan X, Hou X, Li D, Zhang J, Yang W, Qian B, Wu Y, et al. Apatinib Inhibits Cell Proliferation and Induces Autophagy in Human Papillary Thyroid Carcinoma via the PI3K/Akt/mTOR Signaling Pathway. Front Oncol. 2020; 10:217. 10.3389/fonc.2020.00217 32219060 PMC7078169

[23] Zheng B, Ren T, Huang Y, Guo W. Apatinib inhibits migration and invasion as well as PD-L1 expression in osteosarcoma by targeting STAT3. Biochem Biophys Res Commun. 2018; 495:1695–701. 10.1016/j.bbrc.2017.12.032 29225166

[24] Chen JS, Huang JQ, Luo B, Dong SH, Wang RC, Jiang ZK, Xie YK, Yi W, Wen GM, Zhong JF. PIK3CD induces cell growth and invasion by activating AKT/GSK-3β/β-catenin signaling in colorectal cancer. Cancer Sci. 2019; 110:997–1011. 10.1111/cas.13931 30618098 PMC6398891

[25] Ferlay J, Soerjomataram I, Dikshit R, Eser S, Mathers C, Rebelo M, Parkin DM, Forman D, Bray F. Cancer incidence and mortality worldwide: sources, methods and major patterns in GLOBOCAN 2012. Int J Cancer. 2015; 136:E359–86. 10.1002/ijc.29210 25220842

[26] Oba K, Paoletti X, Bang YJ, Bleiberg H, Burzykowski T, Fuse N, Michiels S, Morita S, Ohashi Y, Pignon JP, Rougier P, Sakamoto J, Sargent D, et al, and GASTRIC (Global Advanced/Adjuvant Stomach Tumor Research International Collaboration) Group. Role of chemotherapy for advanced/recurrent gastric cancer: an individual-patient-data meta-analysis. Eur J Cancer. 2013; 49:1565–77. 10.1016/j.ejca.2012.12.016 23352439

[27] Wagner AD, Grothe W, Haerting J, Kleber G, Grothey A, Fleig WE. Chemotherapy in advanced gastric cancer: a systematic review and meta-analysis based on aggregate data. J Clin Oncol. 2006; 24:2903–09. 10.1200/JCO.2005.05.0245 16782930

[28] Lordick F, Allum W, Carneiro F, Mitry E, Tabernero J, Tan P, Van Cutsem E, van de Velde C, Cervantes A. Unmet needs and challenges in gastric cancer: the way forward. Cancer Treat Rev. 2014; 40:692–700. 10.1016/j.ctrv.2014.03.002 24656602

[29] Bang YJ, Van Cutsem E, Feyereislova A, Chung HC, Shen L, Sawaki A, Lordick F, Ohtsu A, Omuro Y, Satoh T, Aprile G, Kulikov E, Hill J, et al, and ToGA Trial Investigators. Trastuzumab in combination with chemotherapy versus chemotherapy alone for treatment of HER2-positive advanced gastric or gastro-oesophageal junction cancer (ToGA): a phase 3, open-label, randomised controlled trial. Lancet. 2010; 376:687–97. 10.1016/S0140-6736(10)61121-X 20728210

[30] Fuchs CS, Tomasek J, Yong CJ, Dumitru F, Passalacqua R, Goswami C, Safran H, Dos Santos LV, Aprile G, Ferry DR, Melichar B, Tehfe M, Topuzov E, et al, and REGARD Trial Investigators. Ramucirumab monotherapy for previously treated advanced gastric or gastro-oesophageal junction adenocarcinoma (REGARD): an international, randomised, multicentre, placebo-controlled, phase 3 trial. Lancet. 2014; 383:31–39. 10.1016/S0140-6736(13)61719-5 24094768

[31] Peng W, Zhang F, Wang Z, Li D, He Y, Ning Z, Sheng L, Wang J, Xia X, Yu C, Wang Z, Zhao Y, Liang H, et al. Erratum: Large Scale, Multicenter, Prospective Study of Apatinib in Advanced Gastric Cancer: A Real-World Study from China [Corrigendum]. Cancer Manag Res. 2020; 12:8409. 10.2147/CMAR.S279084 32821164 PMC7418160

[32] Xi Y, Niu J, Li D, He J, Qin L, Peng X. Mixed lineage kinase-4 promotes gastric carcinoma tumorigenesis through suppression of the c-Jun N-terminal kinase signaling pathway. Exp Ther Med. 2018; 16:3317–24. 10.3892/etm.2018.6618 30233678 PMC6143876

[33] Lian G, Li L, Shi Y, Jing C, Liu J, Guo X, Zhang Q, Dai T, Ye F, Wang Y, Chen M. BI2536, a potent and selective inhibitor of polo-like kinase 1, in combination with cisplatin exerts synergistic effects on gastric cancer cells. Int J Oncol. 2018; 52:804–14. 10.3892/ijo.2018.4255 29393385 PMC5807034

[34] Baghery Saghchy Khorasani A, Pourbagheri-Sigaroodi A, Pirsalehi A, Safaroghli-Azar A, Zali MR, Bashash D. The PI3K/Akt/mTOR signaling pathway in gastric cancer; from oncogenic variations to the possibilities for pharmacologic interventions. Eur J Pharmacol. 2021; 898:173983. 10.1016/j.ejphar.2021.173983 33647255

[35] Jiang H, Zhou Z, Jin S, Xu K, Zhang H, Xu J, Sun Q, Wang J, Xu J. PRMT9 promotes hepatocellular carcinoma invasion and metastasis via activating PI3K/Akt/GSK-3β/Snail signaling. Cancer Sci. 2018; 109:1414–27. 10.1111/cas.13598 29603830 PMC5980302

[36] Wei B, Wang Y, Wang J, Cai X, Xu L, Wu J, Wang Y, Liu W, Gu Y, Guo W, Xu Q. Apatinib suppresses tumor progression and enhances cisplatin sensitivity in esophageal cancer via the Akt/β-catenin pathway. Cancer Cell Int. 2020; 20:198. 10.1186/s12935-020-01290-z 32514243 PMC7254695

[37] Cheng J, Wang Y, Zhang CF, Wang H, Wu WZ, Pan F, Hong N, Deng J. Chemotherapy response evaluation in a mouse model of gastric cancer using intravoxel incoherent motion diffusion-weighted MRI and histopathology. World J Gastroenterol. 2017; 23:1990–2001. 10.3748/wjg.v23.i11.1990 28373765 PMC5360640

[38] Zhang C, Awasthi N, Schwarz MA, Hinz S, Schwarz RE. Superior antitumor activity of nanoparticle albumin-bound paclitaxel in experimental gastric cancer. PLoS One. 2013; 8:e58037. 10.1371/journal.pone.0058037 23460921 PMC3584019

[39] Feitsma H, Cuppen E. Zebrafish as a cancer model. Mol Cancer Res. 2008; 6:685–94. 10.1158/1541-7786.MCR-07-2167 18505914

[40] Payne E, Look T. Zebrafish modelling of leukaemias. Br J Haematol. 2009; 146:247–56. 10.1111/j.1365-2141.2009.07705.x 19466976

[41] Lee LM, Seftor EA, Bonde G, Cornell RA, Hendrix MJ. The fate of human malignant melanoma cells transplanted into zebrafish embryos: assessment of migration and cell division in the absence of tumor formation. Dev Dyn. 2005; 233:1560–70. 10.1002/dvdy.20471 15968639

[42] Veinotte CJ, Dellaire G, Berman JN. Hooking the big one: the potential of zebrafish xenotransplantation to reform cancer drug screening in the genomic era. Dis Model Mech. 2014; 7:745–54. 10.1242/dmm.015784 24973744 PMC4073264

